# Utilising artificial intelligence to identify surgical anatomy during laparoscopic donor nephrectomy – a validation and feasibility study

**DOI:** 10.1038/s41598-026-35999-0

**Published:** 2026-02-05

**Authors:** Chloe Shu Hui Ong, Hoi Pong Nicholas Wong, Manchi Leung, Yu-Chieh Lee, Bo-An Tsai, Seu-Hwa Chen, Jeff Shih-Chieh Chueh, Ho Yee Tiong

**Affiliations:** 1 Department of Urology, National University Hospital (S) Pte Ltd, National University Health System, 1E Kent Ridge Road, NUHS Tower Block, Level 8, Singapore, 119228 Singapore; 2 Smart Surgery Technology Co. Ltd, Taipei, Taiwan; 3 Department of Anatomy and Cell Biology, School of Medicine, College of Medicine, Taipei Medical University, Taipei, Taiwan; 4 Department of Urology, National Taiwan University Hospital, College of Medicine, National Taiwan University, Taipei, Taiwan; 5 Department of Surgery, National University of Singapore, Singapore, Singapore

**Keywords:** Nephrectomy, Donor nephrectomy, Artificial intelligence, Intra-operative guidance, Anatomy, Computational biology and bioinformatics, Health care, Medical research, Urology

## Abstract

Although the risk of intraoperative complications of laparoscopic donor nephrectomy (LDN) is now acceptably low, the work continues to minimise technical mishaps during this ‘high stakes’ surgery. In this study, we aim to demonstrate the pilot use of a patented proprietary deep learning (DL)-based computer vision (CV) to automatically recognise key anatomical structures and prevent intraoperative injuries, which is especially crucial during the learning curve. 6828 images manually annotated by pixels were selected from 16 surgical videos (National University Hospital, NUH) for training as ground truth, and 1757 annotated images from 4 separate surgical videos were used for validation. This ensured a balanced validation ratio of nearly 20% for each label (spleen, left kidney, renal artery, renal vein, and ureter). The YOLO (you only look once) v11x DL network (https://docs.ultralytics.com/models/yolo11/), known for its speed and accuracy in real-time detection, was adapted to train our model. For further optimisation, it uses a sophisticated loss function which incorporates the accuracy of each pixel in segmentation tasks (binary cross-entropy loss), compares the predicted bounding box coordinates against ground truth (bounding box loss), and emphasises the importance of difficult-to-detect labels (distribution focal loss). Metrics were calculated using the following formulas, based on true positives (TP), false positives (FP), and false negatives (FN): Precision: TP / (TP + FP), Recall: TP / (TP + FN), F1 Score: 2 * (Precision * Recall) / (Precision + Recall). High precision minimises false positives, which could disrupt surgical workflows, while high recall ensures comprehensive detection, minimising false negatives that could affect patient safety. F1 serves as the harmonic mean of recall and precision. Quantitative evaluation of the validation dataset using the hold-out validation method yielded promising performance metrics and prospective evaluation was performed on a video from another surgeon (JC) and institution (National Taiwan University) and also in real-time in NUH. Our pilot study demonstrates an innovative machine learning design’s ability to accurately identify vital anatomical structures in LDN. This is a crucial first step for further artificial intelligence (AI)-guided applications such as intra-operative guidance, education, and post-hoc operative analysis and operative standards evaluation.

## Introduction

Artificial intelligence (AI) and computer vision (CV) have rapidly advanced over the past decade, enabling automated analysis of intra-operative videos and real-time identification of surgical phases, instruments, and anatomical structures^1–4^. Within the realm of machine learning, deep learning (DL) underpins the majority of these advances by enabling complex pattern recognition and feature extraction from large volumes of surgical data. In urological surgery, these technologies have been leveraged to augment performance, improve intra-operative guidance, and potentially reduce technical errors^5–7^.

The integration of AI into minimally-invasive and robotic nephrectomy has facilitated the development of augmented reality (AR) navigation, automated 3D model overlays, and real-time instrument tracking, thereby improving surgical precision and workflow efficiency. Recent studies have demonstrated the feasibility of AI-driven AR systems for automatic registration of virtual models with intra-operative anatomy, reducing the reliance on manual alignment, which results in more accurate and precise localization of critical structures such as the hilum^5,6,8^. These innovations are complemented by advanced instrument detection and tracking frameworks that have shown high accuracy and speed in real-time laparoscopic environments, minimizing errors and cognitive loads^1,9^.

Despite these developments, the clinical adoption of intra-operative AI remains in its infancy, with current systems operating at low levels of autonomy and requiring further validation in prospective human studies^10,11^. These developments are also increasingly recognized as transformative tools in surgical training and assessment^12,13^. By providing objective and automated feedback on surgical performance, there is less subjectivity – facilitating standardized and reproducible assessments across trainees^14^.

Laparoscopic donor nephrectomy (LDN) patients are altruistic volunteers who do not need surgery; and hence it is considered a ‘high stakes’ procedure for which it is imperative that intra-operative complications are minimized. Since it is a relatively straightforward operation in healthy individuals, this surgery is ideal for the initial incorporation of AI in recognizing anatomical structures and key surgical steps.

In this paper, we present a proof-of-concept and feasibility study demonstrating the pilot use of a patented proprietary DL-based CV system to automatically recognise key anatomical structures during the operation. This can subsequently translate into other AI-guided applications such as intra-operative guidance, education, and post-hoc operative analysis and operative standards evaluation. Given the established nature of LDN within urological practice, this manuscript focuses primarily on the feasibility and clinical application of AI-based anatomical recognition.

## Materials and methods

### Computer vision system

A deep learning-based computer vision algorithm, developed by Smart Surgery Technology (Taiwan), employs instance segmentation techniques within the Holoscan graph-based operator imaging processing pipeline. This facilitates the real-time, automatic identification of anatomical structures, including the kidney, spleen, renal artery, renal vein, and ureter. The machine learning model was trained and validated using Nvidia A10G with 4 Tensor Core GPUs, 96 vCPUs, and 384 GiB of memory, powered by AMD EPYC processors. Real time inference was performed by Nvidia IGX Orin embedded device with 248 TOPs performance.

### Data collection and pre-processing

Thirty anonymised surgical videos of left LDN, in MP4 format with a display resolution of 1080 pixels and a frame rate of 30 frames per second, were collected retrospectively from 2018 to 2023 within our institution (National University Hospital, Singapore). Institutional Review Board (IRB) approval was obtained for this study (2025/1132). As only fully de-identified intraoperative videos without any patient information were used, the requirement for informed consent was waived. All methods were carried out in accordance with the relevant guidelines and regulations. We focused specifically on the post-dissection stage where the surgeon had already removed the fat and surrounding tissue leaving the renal artery, renal vein, and ureter clearly exposed. Representative frames were extracted and segmented into static image units saved for offline quantitative analysis.

To standardize the input data for the real-time AI model, each image was resized to 640 × 640 pixels. Additionally, the intensity levels of the images were normalized using standard deviation normalization, such that the pixel values were adjusted to a mean of 0 and a standard deviation of 1. These pre-processing steps ensured consistency in image size and pixel intensity, hence improving the model’s ability in identifying key structures during the post-dissection phase of surgery.

**Fig. 1:**
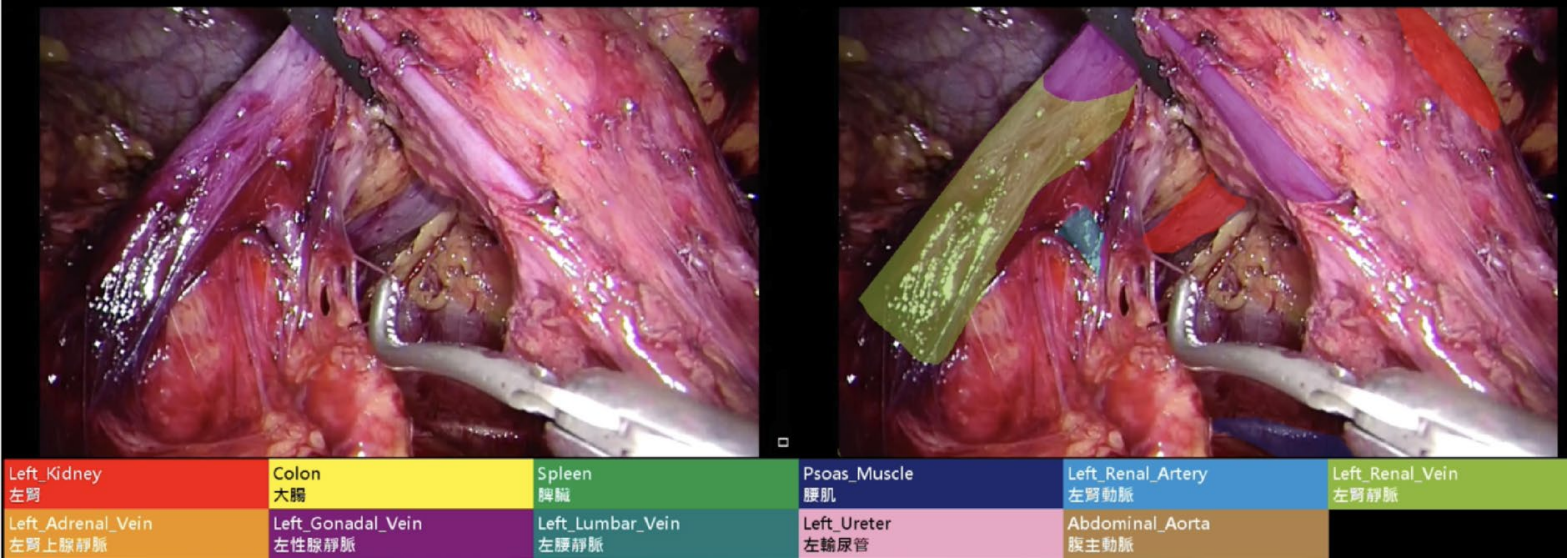
Demonstration of static image unit (left) and labelled images (right).

Each image was then individually analysed with pixels corresponding to anatomical targets manually labelled by an academic anatomist (S-H. C.) and subsequently reviewed for accuracy thereafter by an American Society of Transplant Surgeons (ASTS)-accredited surgeon (H. Y. T.) as part of a dual-review quality assurance process (Fig. 1). The key relevant anatomical targets of concern include the following: left kidney, left renal artery, left renal vein, left adrenal vein, left gonadal vein, left lumbar vein, left ureter, colon, spleen, psoas muscle, and the abdominal aorta. These annotated images were then used to train the AI model.

The surgical videos used for training and validation were distinct. A total of 6828 images from 16 videos were used for training, encompassing both positive and negative examples. For testing, 1757 images were extracted from 4 separate surgical videos to validate and assess the DL software. The dataset was split approximately 80:20, with the label distribution maintained across both the training and validation sets.

In addition, to ensure that the training dataset is representative, we implemented a selective data inclusion strategy. This strategy allowed us to include images representing less frequent but critical anatomical structures.

### Model architecture and training

The You Only Look Once (YOLO) v11x network, known for its speed and accuracy in real-time detection, was adapted to train our model to identify anatomical structures with high precision and recall. This is a well-established model that excels in detecting objects in a single pass, making it ideal for applications such as in laparoscopic surgery (https://ieeexplore.ieee.org/document/7780460). The YOLO v11 network incorporates a multi-head attention mechanism in its backbone, allowing it to capture fine details such as the renal hilar structures. Additionally, it employs depthwise convolution to reduce parameter requirements, resulting in a more efficient training process. For our study, we utilized the v11x high-performance version to address the challenge of detecting the often small and partially obscured structures. The model was optimized to operate on high-performance hardware. During training, it utilized Nvidia A10G GPUs with Tensor Core capabilities to handle the large volume of video data. For real-time inference, the model was deployed on the Nvidia IGX Orin embedded system, which boasts significant computational power (248 TOPs). This hardware setup allows the model to process video data in real time, providing surgeons with timely guidance during critical moments of surgery.

**Fig. 2:**
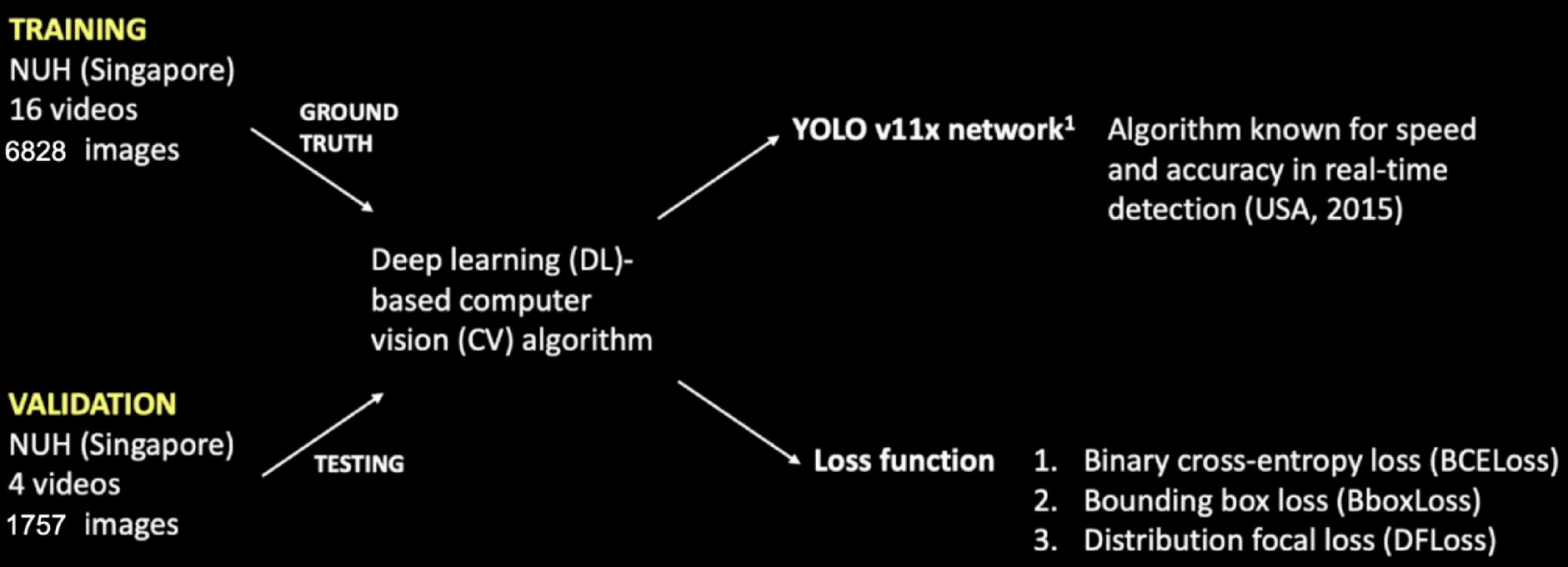
Workflow of data.

To ensure our dataset was representative of key surgical structures, we applied variable importance through a weight tensor of [1, 1, 3, 3, 6] to [spleen, kidney, renal artery, renal vein, ureter] where higher weights were assigned to critical structures in LDN such as the left renal artery, left renal vein, and left ureter. This weighting influenced both loss computation and model validation. Further optimization employed a combination of the following loss functions: (a) binary cross-entropy losses (BCEloss) which measures accuracy of each pixel in segmentation tasks, (b) bounding box losses (BboxLoss) which compares the predicted bounding box coordinates against ground truth and (c) distribution focal loss (DFloss) which emphasizes the importance in difficult-to-detect labels (Fig. 2). By incorporating the weight tensor into the BCEloss calculation, we were able to emphasize the importance of difficult-to-detect labels during training, improving the model’s performance in challenging cases. The model was trained for 500 epochs using a batch size of 36. An early stopping mechanism was employed to avoid overfitting, and halting training if validation loss did not improve for 100 consecutive epochs. The Adam (Adaptive Moment Estimation) optimizer was used for gradient-based optimization, with a decaying learning rate schedule.

### Model evaluation

When evaluating a YOLO model after training it on a dataset, the mAP50 (mean Average Precision at IoU threshold 0.5) score is used to summarize its detection performance by evaluating how well the model detects objects with reasonable localization (50% overlap), and providing a balance between detection accuracy and localization precision.

### Model tuning

We employed Optuna, a robust optimization framework that utilizes Bayesian optimization, to effectively tune our model’s hyperparameters. This method balances exploration and exploitation, facilitating quicker convergence to optimal solutions compared to traditional techniques such as grid search. Our optimization focused on maximizing a fitness metric, defined as a linear combination of evaluation metrics mAP50 and mAP50-95, with weights of [0.2, 0.8].

This approach enabled us to pinpoint the most effective trials for the hyperparameters of our DL model, enhancing its ability to recognize key anatomical structures in real-time surgical environments.

### Quantitative evaluation

#### Confusion matrix

The confusion matrix shows the performance of a YOLO-based segmentation model for different classes related to urological anatomy, such as the spleen, left kidney, left renal artery, left renal vein, left ureter, and background. Each cell in the matrix represents the number of predictions made by the model. The rows correspond to the predicted classes, and the columns correspond to the true classes.

#### Quantitative analysis

Quantitative metrics, such as precision, recall, and F1 score, were calculated from true positives and false negatives. Precision is defined as true positives over the sum of true and false positives, while recall is defined as true positives over the sum of true positives and false negatives. A higher false positive rate can occur when pixels that do not correspond to the renal artery, renal vein, and ureters are incorrectly identified, resulting in lower precision. Conversely, a higher false negative rate can occur when pixels corresponding to the renal artery, renal vein, or ureters are not recognized, leading to lower recall.

F1 score is the harmonic mean of precision and recall, and is defined as two times of the product of precision and recall over the sum of precision and recall. This is a widely-used metric in machine learning to evaluate a model’s performance by combining both precision and recall into a single value, giving more weight to situations whereby both metrics are high and penalizing large discrepancies between them^15^. A score of more than 0.4 is generally accepted in real-time detection.

### Further analyses

Further prospective analyses was performed by running the developed algorithm on two LDN videos from another surgeon and institution (National Taiwan University), and also on-site validation during a real-time LDN in NUH.

## Results

**Fig. 3:**
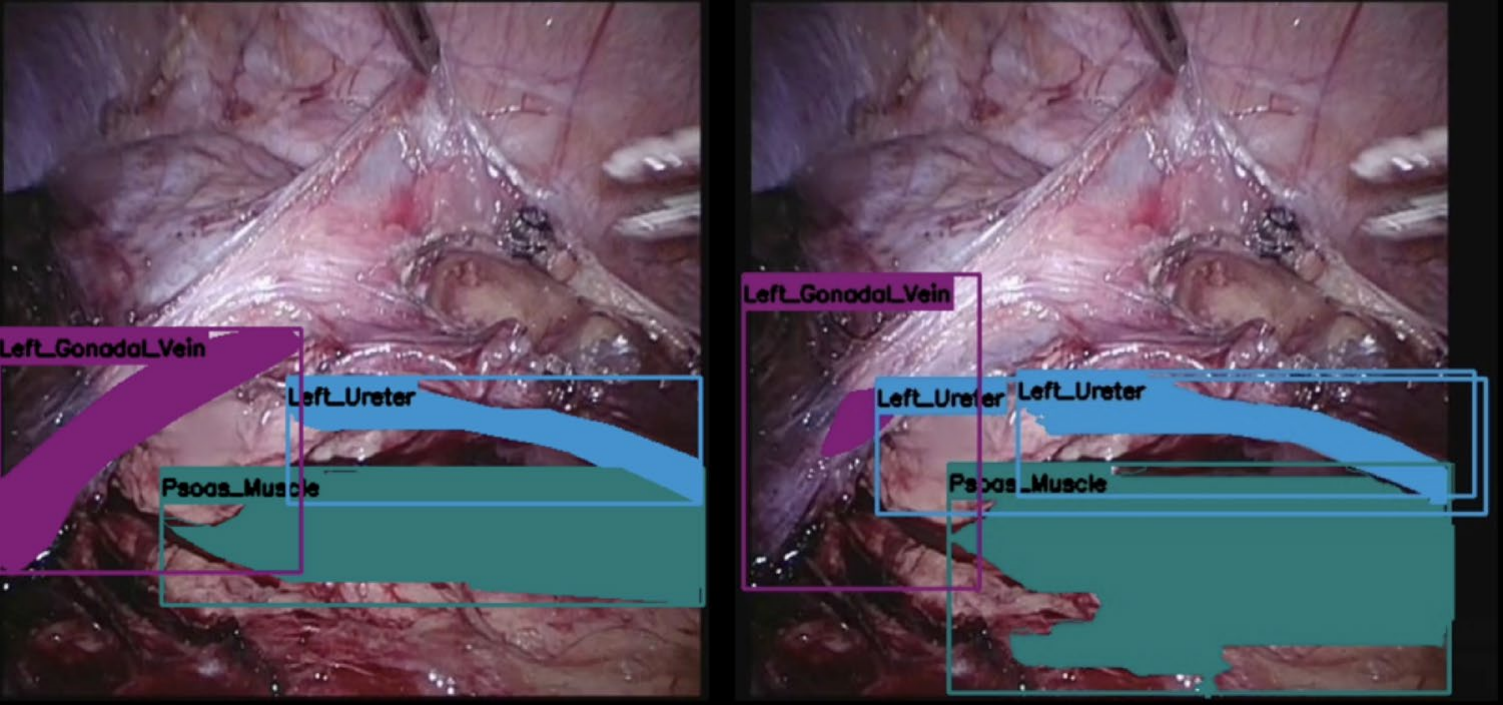
Manually annotated image (left) and AI produced annotations (right).

**Fig. 4:**
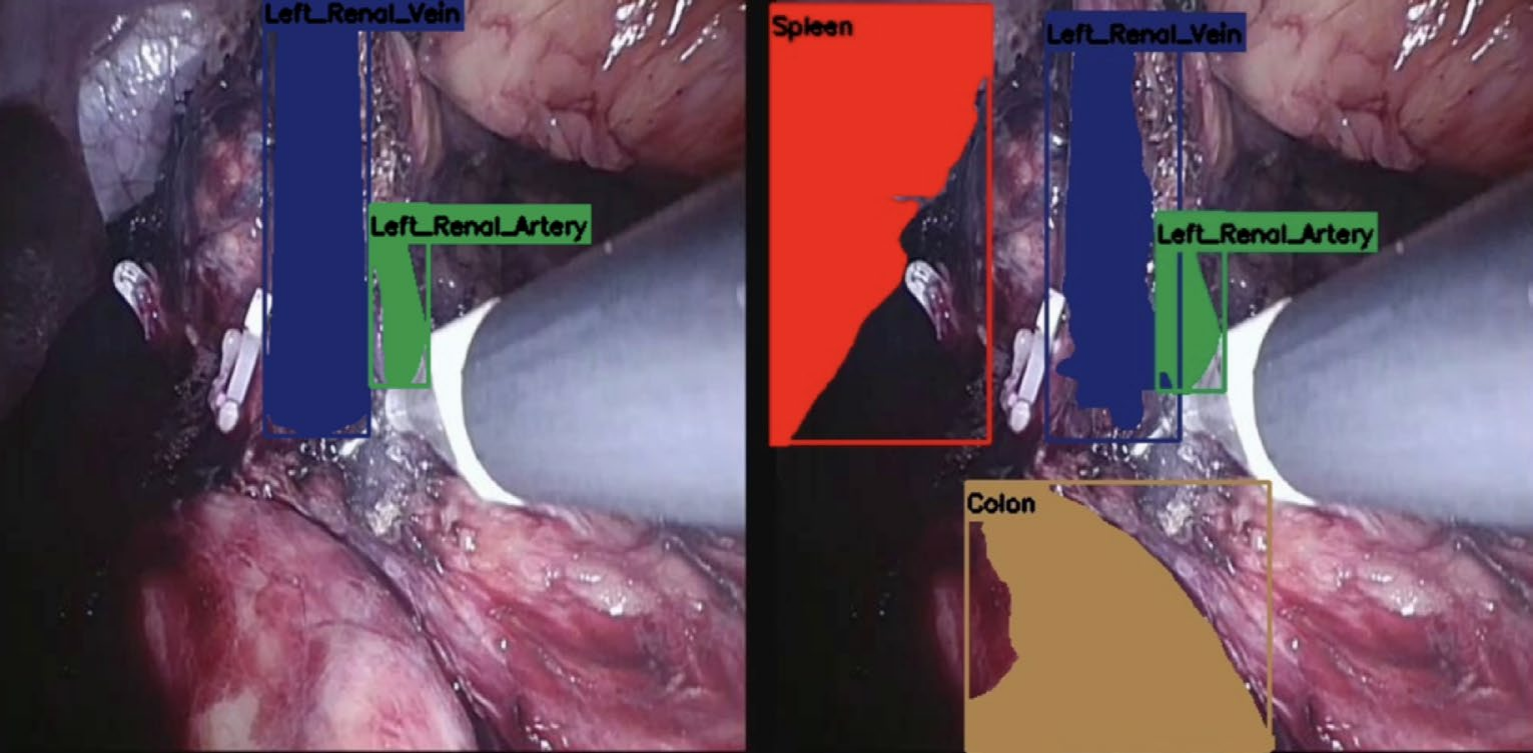
Manually annotated image (left) and AI produced annotations (right).

**Fig. 5:**
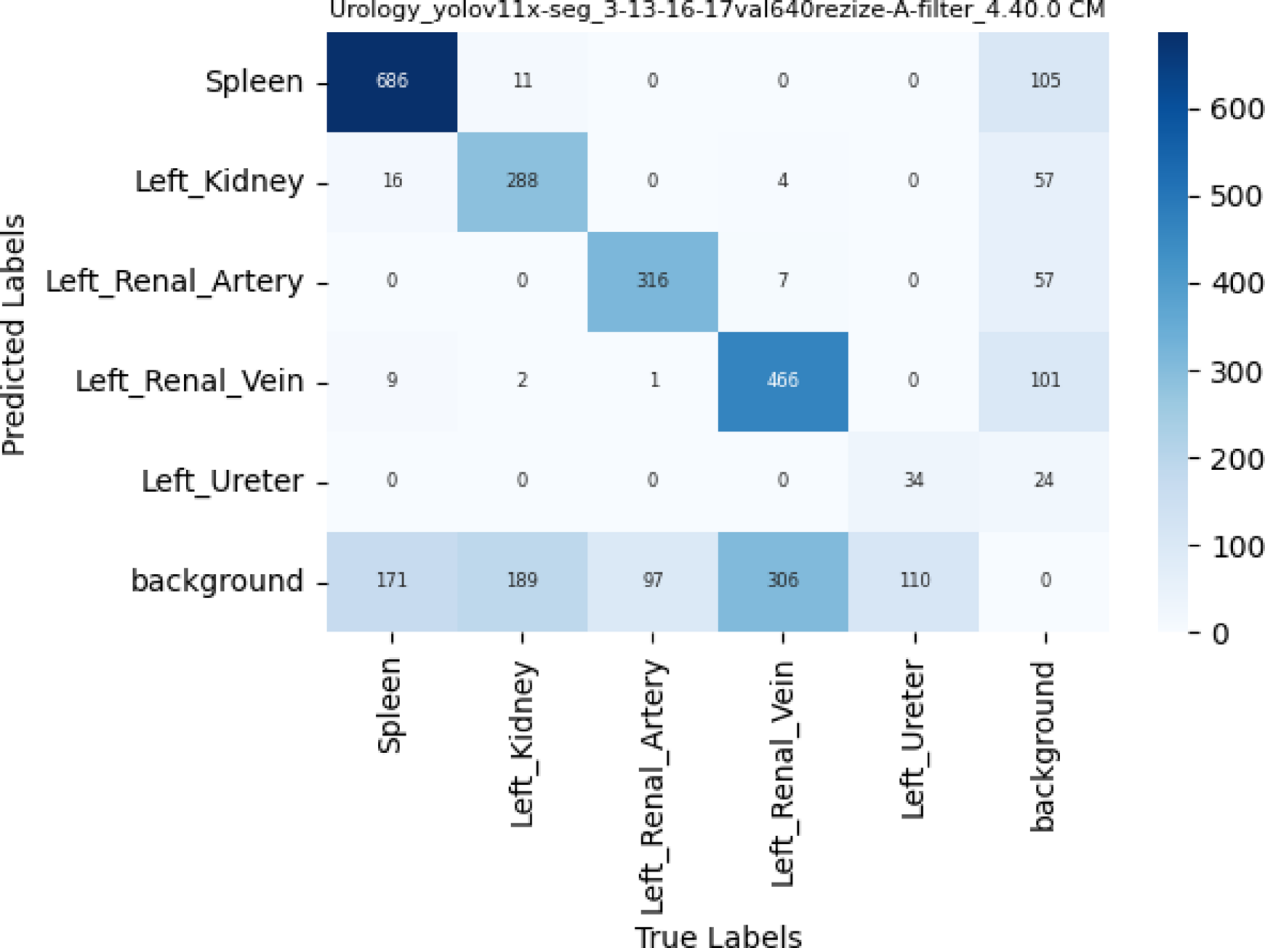
Confusion matrix, demonstrating predicted labels against true labels.

Following initial training using the 16 ground truth videos, the DL network was tested against 4 separate surgical videos containing 1757 images (Figs. 3 and 4). The manually labelled and annotated images were compared against the automatically generated images. The precision, recall, and F1 score of the various key anatomical structures seen intraoperatively were consequently attained (Table 1). The confusion matrix was also attained in (Fig. 5). The automatic software demonstrated encouraging performance in identifying the spleen and the hilar structures. The highest precision was in identifying the spleen, whilst the highest recall was both in the spleen and in the renal artery. The spleen also scored highest in the F1 score. However, segmentation performance was notably weaker for the ureter. This could be due to the mobile nature of the ureter, the overall small width of the ureter, or even possibly reflecting class imbalance and limited representation within the training dataset.

**Table 1:** Precision, recall and F1 scores comparing artificial intelligence against ground truth.

Class	Precision	Recall	F1 score
Spleen	0.86	0.76	0.88
Left kidney	0.78	0.58	0.67
Left renal artery	0.83	0.76	0.79
Left renal vein	0.78	0.59	0.68
Left ureter	0.55	0.23	0.32

**Fig. 6:**
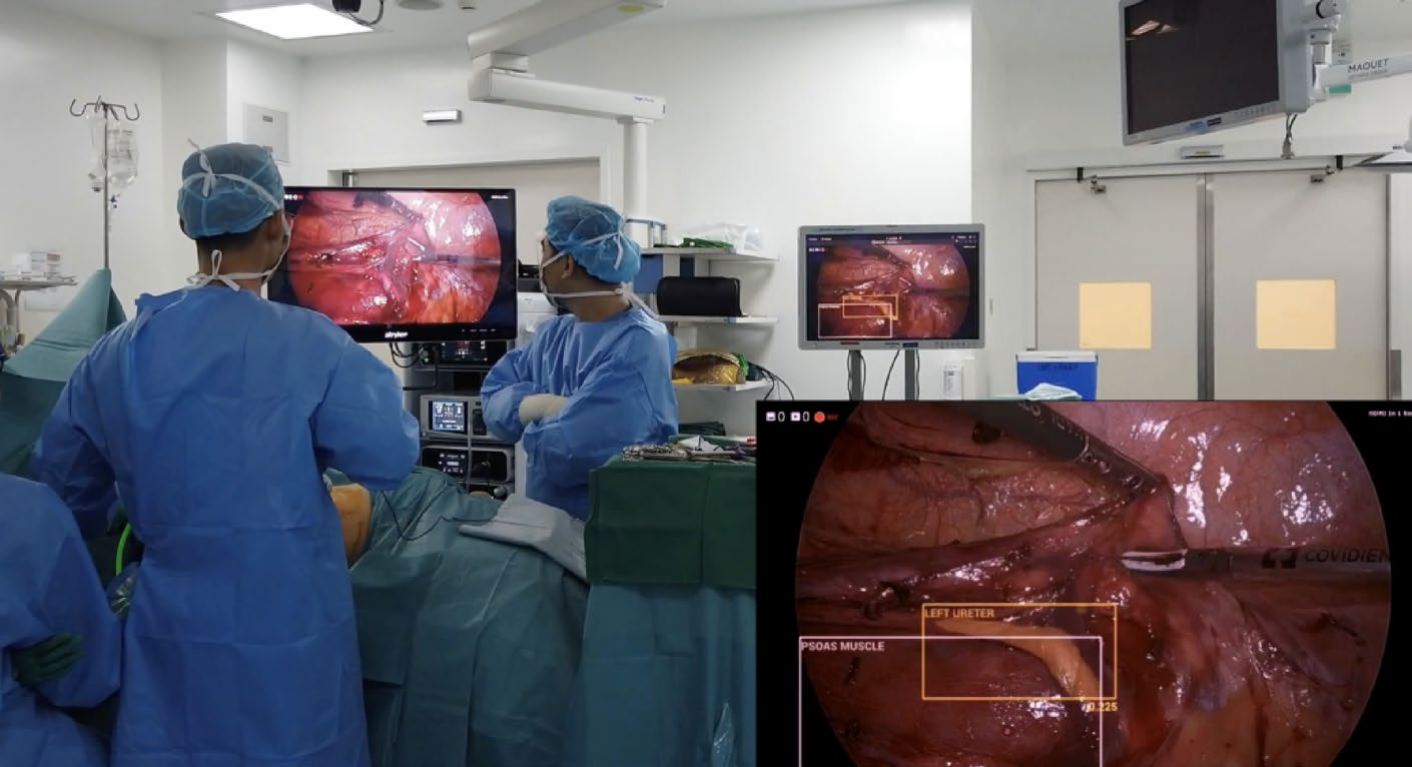
Intraoperative real-time use of the artificial intelligence.

**Fig. 7:**
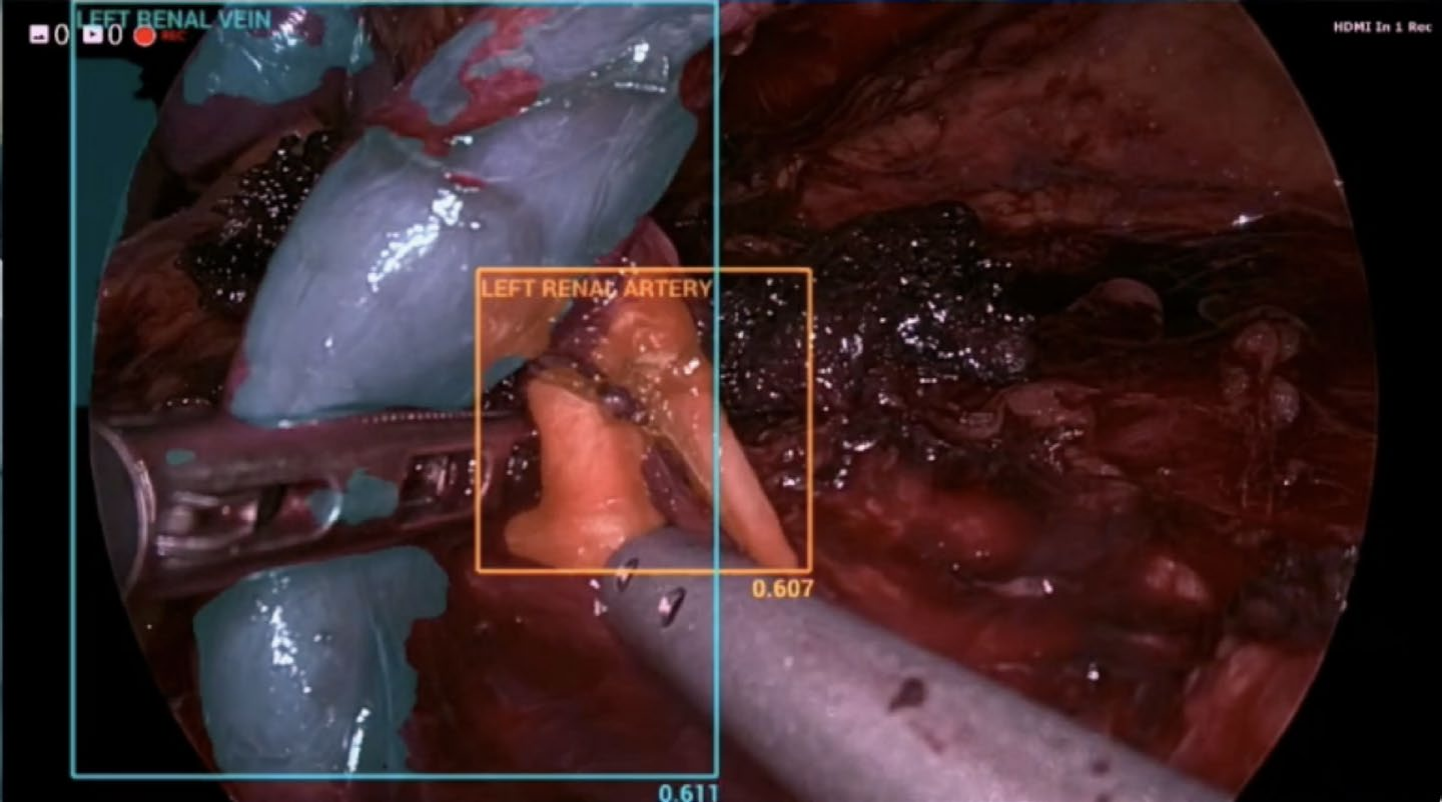
Dual renal artery identified by AI.

In addition to offline quantitative evaluation, the model was subjectively demonstrated in real-time during a laparoscopic donor nephrectomy to assess its ability to identify intraoperative anatomical structures in real time (Fig. 6). Alongside identifying conventional anatomy, the model was also observed to recognize anatomical anomalies such as dual artery during real-time demonstration (Fig. 7).

## Discussion

The use of semantic segmentation to identify anatomical structures with AI has been reported in laparoscopic cholecystectomy and colorectal surgery^16,17^. To the authors’ knowledge, this is among the first studies to apply real-time AI-based anatomical segmentation in urological surgery, building upon prior success and work in other surgeries.

Quantitative metrics from our feasibility study have yielded encouraging preliminary results. Precision scores for the hilar structures such as the left kidney (0.78), left renal artery (0.83), and left renal vein (0.78) are high – this minimizes false positives which could disrupt surgical workflow. Recall scores are also high for the left kidney (0.58), left renal artery (0.76), and left renal vein (0.59), which minimizes false negatives and ensures patient safety. The F1 score is consequently > 0.4 (generally accepted cut-off in real-time detection) for these structures. On the other hand, the left ureter did not perform as well with a precision score of 0.55, recall score of 0.23, and F1 score of 0.32. This is likely due to the ureter being thin and similar in colour to its surroundings. As such, more videos are required to better train the algorithm to identify the ureter accurately.

This study is not without its challenges. Firstly, the raw data used to train the model is limited. We have only used images from 16 videos originating from a single institution as the training dataset – this was a carefully considered decision for our proof-of-concept study in order to train the initial algorithm more efficiently. However, we acknowledge that this limits external validity given known inter-centre variability in optics, surgical approach, lighting conditions and video quality, amongst others. To address that, we ran the algorithm against videos from another institution and country, and showed that it was still able to perform well. In order to make the algorithm more robust, the next step would be to include videos from other institutions and surgeons in the training and validation dataset, although this increased variability may adversely affect the precision, recall and F1 scores. Additionally, institutional practices, surgeon-specific techniques and patient-related factors such as body habitus, the presence of inflammatory changes and intraoperative bleeding can obscure the visibility of critical anatomical structures, introducing bias and variability. This further limits the generalizability of the current model. These factors may complicate the model’s task of distinguishing between similar-looking tissues, leading to potential misclassifications. The current model also lacks a spatial reference priori, which means that it does not consider the known relative positions of anatomical structures. This limitation may hinder the model’s accuracy as organs may constantly change during surgery. As such, the addition of a spatial correlation framework for reference can refine the algorithm instead of relying solely on visual cues, which may be insufficient in more complex cases.

This model is still in its infancy and not yet at the stage of routine intra-operative guidance. While real-time anatomical recognition was subjectively demonstrated, formal performance metrics such as latency measurements, frame-to-frame consistency and failure analyses were not assessed and represent a limitation of the present study. However, the identification of key anatomical structures is an essential first step for further AI-guided applications, particularly in clinical scenarios such as surgical training, recognition of anatomical variants and intra-operative situational awareness during high-risk dissection phases. For example, in the training of surgical trainees, videos can be retrospectively or intra-operatively annotated by AI to demonstrate important structures to trainees, which would aid in increased exposure to both conventional and variant anatomies. Secondly, experienced surgeons can similarly benefit from this new technology in the form of post-hoc operative analyses and evaluation of recorded videos, which can automatically generate objective performance metrics instead of depending on subjective opinions of what a good surgery entails^18^. Both of these will hopefully translate into earlier mounting of learning curves and accelerate the learning process cost-effectively. Kolbinger et al. have demonstrated that AI models consistently outperformed medics of various levels of seniority in the identification of pancreas anatomy^19^, hence supporting the utility of AI assistance in surgery.

Future prospects of AI-guided applications include combining real-time surgical videos with 3-dimensional models generated from CT scans, alongside formal evaluation of real-time performance characteristics. This will give surgeons a better idea of both surface and internal anatomy and undoubtedly enhance accuracy; or even correlating intra-operative images with final histology for early diagnoses^20^.

## Conclusion

In this study, we present the pilot use of an innovative machine learning design’s ability to accurately identify vital anatomical structures in LDN, with high precision, recall and F1 scores, especially in the hilar area. Additionally, we have demonstrated its transferability on another institution’s recorded video, and also intra-operatively in real-time in our institution. This is a crucial first step for further AI-guided applications such as intra-operative guidance, education, and post-hoc operative analysis and operative standards evaluation. As such, further training and validation of the model is required for continued improvement of the precision, recall, and F1 scores.

## Data Availability

All data generated or analysed during the study that are relevant are included in the published paper. The datasets generated during and/or analyzed during the current study are available from the corresponding author on reasonable request.
